# Investigation of Dry Eye Symptoms in Lecturers by Ocular Surface Disease Index

**DOI:** 10.4274/tjo.galenos.2018.67915

**Published:** 2019-06-27

**Authors:** Sümbüle Köksoy Vayısoğlu, Emine Öncü, Özer Dursun, Erdem Dinç

**Affiliations:** 1 Mersin University Faculty of Nursing, Department of Public Health Nursing, Mersin, Turkey; 2 Mersin University Faculty of Medicine, Department of Ophthalmology, Mersin, Turkey

**Keywords:** Dry eye, ocular surface disease index, OSDI, lecturers

## Abstract

**Objectives:**

The aim of this study was to evaluate the prevalence of dry eye symptoms among lecturers.

**Materials and Methods:**

The study included 254 lecturers employed at Mersin University. The lecturers were selected by simple random sampling from lists obtained from the personnel department. Data were obtained between November 15 and December 15, 2017 using a questionnaire developed by the researchers and the Ocular Surface Disease Index (OSDI). The data were evaluated using descriptive statistics, Student’s t test, ANOVA, and correlation tests with the SPSS package program.

**Results:**

Of the lecturers who participated in the study, 52.8% were male and 47.2% were female, and the mean age was 39.29±9.41 years. According to OSDI scores, 20.5% of the participants had mild, 15% had moderate, and 36.5% had severe disease. There were significant differences in mean OSDI score based on sex (p<0.001), alcohol use (p=0.01), continuous drug use (p=0.03), wearing glasses (p=0.04), history of dry eye (p<0.001), and presence of dry eye symptoms (p<0.001). There were also significant differences between the OSDI score categories in terms of sex (p<0.001), smoking status (p=0.04), wearing glasses (p=0.03), history of dry eye (p<0.001), and presence of dry eye symptoms. The only factor significantly correlated with OSDI score was daily duration of computer usage (p=0.009).

**Conclusion:**

Our study showed that a substantial proportion of lecturers experience dry eye symptoms, and OSDI scores were associated with daily duration of computer use. Determining the factors associated with dry eye is important for the planning of preventive interventions.

## Introduction

Dry eye is a multifactorial disease of the ocular surface characterized by loss of tear film homeostasis, in which neurosensory abnormalities play an etiological role.^1^ Accompanied by ocular surface inflammation and damage, dry eye is an important disease that can impair quality of life. According to the DEWS II report, the reported prevalence of dry eye varies between 5% and 50%, with the frequency of signs being higher and more variable compared to symptoms.^2^

The development of dry eye involves two basic mechanisms, excessive tear evaporation and aqueous deficiency. Approximately 10% of patients have aqueous deficiency alone, while more than 80% have both aqueous deficiency and excessive evaporation due to meibomian gland dysfunction (MGD).^3^ There are modifiable and nonmodifiable risk factors associated with these mechanisms of dry eye development. The main nonmodifiable risk factors are age, female sex, Asian race, Sjögren’s syndrome, soft tissue diseases, MGD, androgen deficiency, and the use of certain drugs (e.g., isotretinoin), while modifiable risk factors include intensive computer use, contact lens use, and environmental factors (pollution, low humidity, sick building syndrome, etc.).^2,3,4^ Prolonged use of computers and smartphones, which have become a part of daily life, are major factors contributing to the increased prevalence of dry eye.^5^ Reduced blinking rate when looking at the screen, the type of screen used, and the angle and distance between the eyes and screen can pose a risk for dry eye. Eye fatigue and dry eye syndrome are especially common among individuals who are also exposed to these factors in the workplace.^5,6^ The dry eye diagnosis flowchart begins with history-taking, risk factors are questioned in suspicious cases, and a screening test such as the Ocular Surface Disease Index (OSDI) or Dry Eye Questionnaire is applied. In light of these data, confirming the diagnosis by clinical examination is recommended in necessary cases.^1^

The literature includes previous studies conducted to determine the prevalence of dry eye in different occupational groups that use computers, but we found no study evaluating the prevalence of dry eye among academicians. The aim of the present study was to use the OSDI to determine the incidence of dry eye symptoms among university lecturers. This study is important because it demonstrates that academicians are also at risk of dry eye due to prolonged computer use, and it may facilitate the planning of preventive interventions.

## Materials and Methods

This cross-sectional study was performed between November 15 and December 15, 2017. There were a total of 1615 lecturers working at Mersin University during the study period. Sample size was calculated as 244 people using Epi Info software for a 95% confidence interval and 5% sampling error with an estimated dry eye prevalence of 25%.^1^ The numbers of lecturers were stratified according to school, and the schools to be included in the sample were determined by lottery method. Lecturers were selected using simple random sampling from lists obtained from the personnel department. Those with a history of contact lens use or ocular surgery and those using topical eye drops were excluded. The study data were collected after obtaining ethics committee approval (78017789/050.01.04/478270) and institutional permission. The study was carried out in accordance with the Declaration of Helsinki. Prior to data collection, participants were informed about the study and their consent was obtained. Data collection forms were given in person to those who agreed to participate in the study and collected the next day. Questionnaires were provided to a total of 284 lecturers. After eliminating those with missing data, the questionnaires of a total of 254 lecturers were included in the analysis. The participation rate was 89.4%.

**Figure 1:**
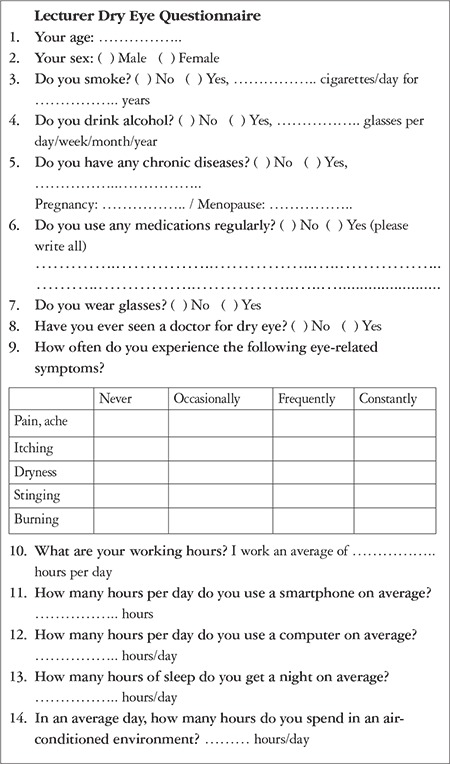
Dry eye questionnaire given to the lecturers

Using a questionnaire developed based on a review of the literature, the lecturers were asked about their socio-demographic characteristics, cigarette/alcohol use, dry eye symptoms, chronic diseases, and medications used, as well as average time per day spent at work, using a computer, smartphone, or tablet, in air-conditioned environments, and sleeping (Figure 1).^5,6,7,8^ Cigarette use was categorized based on the number of cigarettes smoked per day, and alcohol use was categorized by the number of glasses consumed per month. As there were no standards in the literature, daily computer and smartphone use was categorized by 8-hour and 4-hour intervals, respectively. Moreover, systemic drug use was questioned and categorized by drug class.

OSDI scores of 0-12 were classified as normal, 13-22 as mild, 23-32 as moderate, and 33-100 as severe ocular surface disease.^1^ Participants with a score of 13 or higher and those with symptoms of dry eye were considered at risk and referred for eye examination.

### Statistical Analysis

The data were analyzed using SPSS package software. Mean, standard deviation, minimum, and maximum were used for descriptive statistics. Chi-square test was used to analyze categorical variables, correlation analysis was used to evaluate relationships between scores, and mean scores were compared using Student’s t-test and ANOVA. A p value <0.05 was considered significant.

## Results

Of the lecturers included in the study, 52.8% were male, 47.2% were female and the mean age was 39.29±9.41 years (Table 1). Mean time spent at work per day was 8.98±2.15 hours, while the durations of computer and smartphone use were 5.52±2.29 and 2.36±2.50 hours, respectively. The lecturers spent a mean of 7.15±0.99 hours per day in an air-conditioned environment, and their mean sleep duration was 6.85±0.96 hours (Table 2). Categorization of the lecturers based on OSDI score showed that 20.5% had mild, 15% had moderate, and 36.5% had severe ocular surface disease, 52.8% had symptoms of dry eye, and 72.4% experienced symptoms occasionally (Table 2).

Mean OSDI score varied depending on sex (p<0.001), alcohol use (p=0.01), long-term medication use (p=0.03), wearing glasses (p=0.04), previous diagnosis of dry eye (p<0.001), and presence of dry eye symptoms (p<0.001). However, mean OSDI score was not associated with daily activity durations (Table 3).

There were significant differences between OSDI score categories in terms of sex (p<0.001), cigarette use (p=0.04), wearing glasses (p=0.03), previous diagnosis of dry eye (p<0.001), and presence of dry eye symptoms (Table 4). The sex difference was between the normal and severe disease groups, and there was a significant correlation between duration of daily computer use and OSDI score (r=0.164, p=0.009).

## Discussion

A review of the literature shows that some studies evaluating the prevalence of dry eye were based on either symptoms or clinical diagnostic tests, while other studies used both symptoms and clinical signs. Therefore, the outcomes of epidemiological studies vary.^2^ The clinical diagnostic tests used for the diagnosis of dry eye do not always correlate with patients’ symptoms, and the presence of symptoms is important for a preliminary diagnosis of dry eye. In light of this, the main objective of population studies is to identify high-risk individuals and evaluate them using advanced diagnostic methods. The DEWS II report recommended using the OSDI for screening purposes, as this index is considered valid and reliable.^2^ Thus, participants in the present study were assessed with OSDI, and at-risk individuals with OSDI scores over 13 and dry eye symptoms were referred for clinical examination. However, the inability to follow up on the examination findings of the participants is a significant limitation of this study.

Dry eye reduces labor productivity due to its physical effects and time allocated to treatment, can cause psychiatric problems such as depression and anxiety, and can seriously impair sleep quality in some patients.^9,10,11,12^ Therefore, it is important to not overlook the diagnosis, to closely follow patients and control modifiable risk factors, and arrange the necessary treatments.

Globally, the reported incidence of symptomatic or asymptomatic dry eye ranges between 5% and 50%.^2^ Moreover, the frequency of dry eye varies among studies conducted in different geographical regions. In a study conducted in the USA, Farrand et al.^13^ reported the frequency of dry eye among adults over 18 years of age as 6.8%. Unlike these studies, approximately half of the lecturers who participated in our survey reported having at least one symptom of dry eye and three-fourths of the respondents scored 13 or higher on the OSDI, indicating severe disease. These findings are important as an overall indicator that this group is at high risk. The primary factor associated with high risk among the lecturers was prolonged screen time. A meta-analysis by Courtin et al.^14^ showed that the prevalence of dry eye among individuals who used video display terminals (VDTs) for long periods was between 9.5% and 87.5%, with a mean prevalence of 49.5%. In another study by Kawashima et al.^15^, the prevalence of dry eye among workers using VDTs for an average of six hours a day was 60%. Yazici et al.^16^ reported that the incidence of dry eye among individuals who used VDTs for an average of 6.9 hours/day was 27.4%, while this rate was 15.4% among those used VDTs less than an hour per day. Similar to the studies by Kawashima et al.^15^ and Yazici et al.^16^, the average duration of VDT use in our study was nearly 6 hours and 52.8% of participants had symptoms of dry eye. This result seems compatible with the studies in the literature.

The relationship between daily duration of computer use and OSDI scores is known. Gümüş et al.^17^ reported higher OSDI scores among those who used VDTs for an average of 8 hours per day. Simavlı et al.^5^ reported that OSDI scores indicated moderate to severe ocular surface disease in 64% of participants who used a computer for at least 5 hours per day and duration of computer use was positively correlated with OSDI score. Similar results were obtained in another study performed by Büyükbaş et al.^8^, Yazici et al.^16^ and Bayhan et al.^18^ reported significantly higher OSDI scores in those with 7-8 hours of computer use daily compared to those with less than 1 hour per day. Although Akkaya et al.^19^ observed similar OSDI scores in individuals with average daily computer use of 7 hours and less than 1 hour, they noted a difference in their tear breakup times and reported that dry eye developed in the heavier computer users due to excessive tear evaporation. In the current study, half of the participants had OSDI scores indicating moderate/severe ocular surface disease and a significant positive correlation was detected between OSDI score and daily duration of computer use.

In addition to an individual’s daily habits, the physical environment in which they spend their time is important in terms of dry eye development. The DEWS II report stated that the risk of dry eye may increase with the length of time spent in an air-conditioned environment.^2^ Iyer et al.^20^ reported that blurred vision increased with the duration of exposure to air-conditioned environments and could be treated with the use of lubricants, and suggested that this was associated with dry eye. Büyükbaş et al.^8^ found no correlation between air-conditioning and tear volume and function, but emphasized that their findings could not be generalized because the environments in which the measurements were taken were not standardized. In the present study, we observed no significant correlation between OSDI score and length of time spent in an air-conditioned environment. However, similar to the study by Büyükbaş et al.^8^, the accuracy of this finding is uncertain because temperature and humidity of the environment were not measured. In spite of these results, considering the DEWS II report, modifying the physical environments where dry eye patients spend time is recommended. For studies conducted in this context, it is advised to assess the average daily temperature and humidity in workplaces.

Studies have reported that the incidence of dry eye is higher among women and increases with age.^2,14,15^ In the study by Farrand et al.^13^, the prevalence of dry eye was 2.7% in the 18-34 year age group and increased to 18.6% for those 75 or older, and the prevalence was twice as high in women than in men. In another study conducted among Japanese office workers, the prevalence of definite and probable dry eye among women was 76.5% and 60.2% among men. In the same study, it was found that the prevalence of dry eye among those aged 30 or over was 2.22 times higher than in those aged 30 or under.^10^ Consistent with the literature, the prevalence of dry eye symptoms and OSDI scores were significantly higher among the women in our study than the men. However, there was no significant correlation between age and the prevalence of dry eye symptoms. This may be due to the relatively lower mean age of the participants enrolled to our study compared to other studies in the literature.

There is insufficient evidence on the correlation between dry eye and cigarette and alcohol use. Findings of the present study that cigarette use differed between OSDI score categories but OSDI symptom scores did not differ significantly according to cigarette use may be interpreted as evidence that cigarette use exacerbates dry eye symptoms but is not associated with the development of dry eye. However, further studies on this subject are required.

In the current study, mean OSDI score was significantly lower in participants with a history of alcohol use compared to those without. Although data regarding the effect of alcohol use on dry eye development are insufficient, there is evidence suggesting that alcohol increases the symptoms of dry eye.^2^ In the current study, high OSDI scores among participants not using alcohol may be due to them discontinuing alcohol use due to the discomfort it causes, or may be related to the amount of alcohol consumed. Although a meta-analysis suggested that the prevalence of dry eye is 1.15 times higher in alcohol users compared to those who do not use alcohol, it was noted that there may be a false reduction in dry eye prevalence due to the development of peripheral neuropathy in heavy drinkers.^21^ Only present alcohol use was evaluated in our study, and not enough data on lifelong alcohol use were given. The correlation between alcohol and dry eye should be evaluated in different studies.

In the current study, participants with a previous history of dry eye and chronic drug use had higher OSDI scores. Simavlı et al.^5^reported that there was no correlation between OSDI score and the use of glasses. In the present study, it was found that participants who wore glasses had higher OSDI scores than those who did not. An association between dry eye and contact lenses use has been reported in the literature. Lecturers who were actively using contact lenses were excluded from our study, and previous history of contact lens use was not questioned. This finding may stem from the presence of other risk factors independent of wearing glasses. Higher OSDI scores are expected among participants who were previously diagnosed with dry eye and did not receive appropriate and adequate treatment. Certain medications (beta-blockers, diuretics, hormone treatments, anxiolytics) have been reported among the risk factors for dry eye.^2^ In the present study, the medications the participants reported using were consistent with the drugs identified in the literature, which we believe contributed to their dry eye symptoms.

## Conclusion

In conclusion, a significant proportion of lecturers in our sample had dry eye symptoms, and OSDI scores were correlated with daily duration of computer use. This indicates that lecturers are prone to developing dry eye. However, new studies involving more centers and participants should be planned.

## Figures and Tables

**Table 1:**
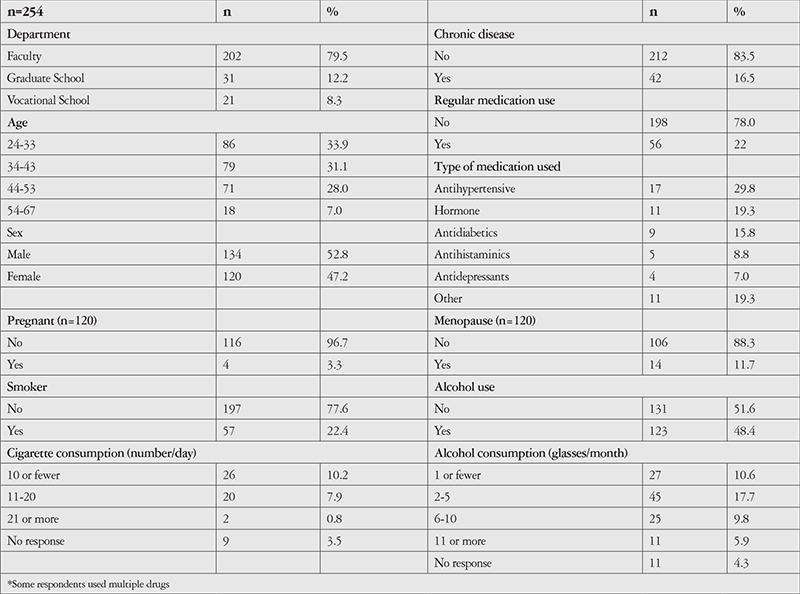
Socio-demographic, clinical, and lifestyle characteristics of the lecturers

**Table 2:**
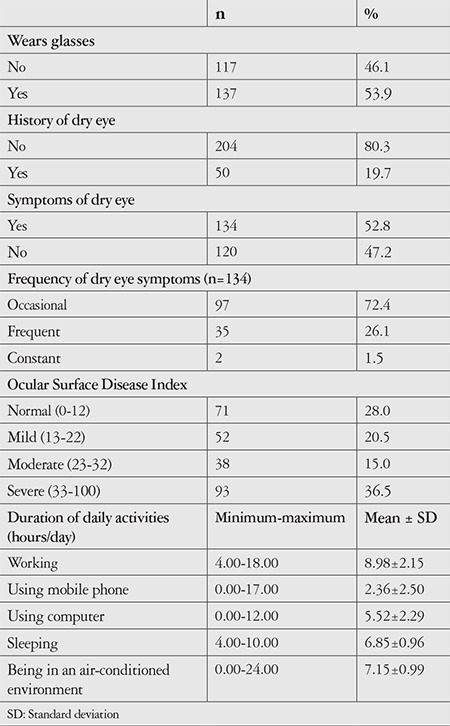
The dry eye symptoms, Ocular Surface Disease Index scores, and daily activity durations of lecturers

**Table 3:**
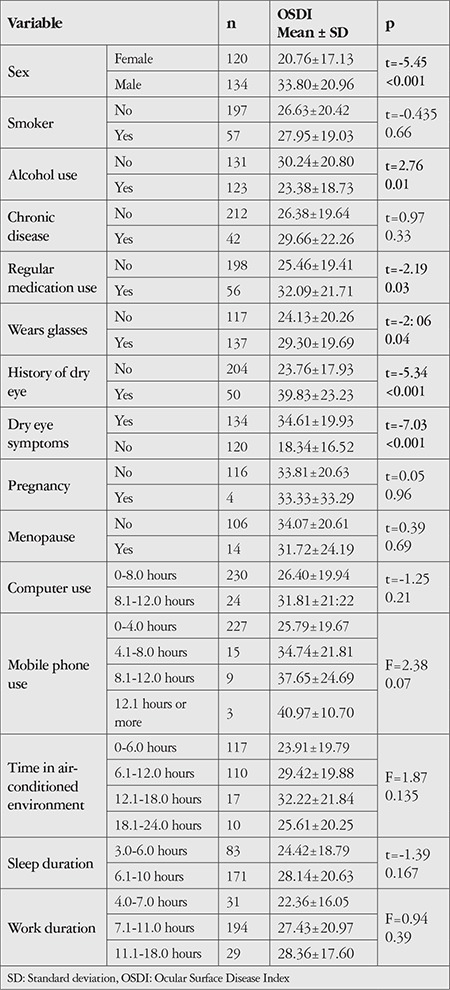
Distribution of mean Ocular Surface Disease Index scores according to demographic characteristics and daily activity durations

**Table 4:**
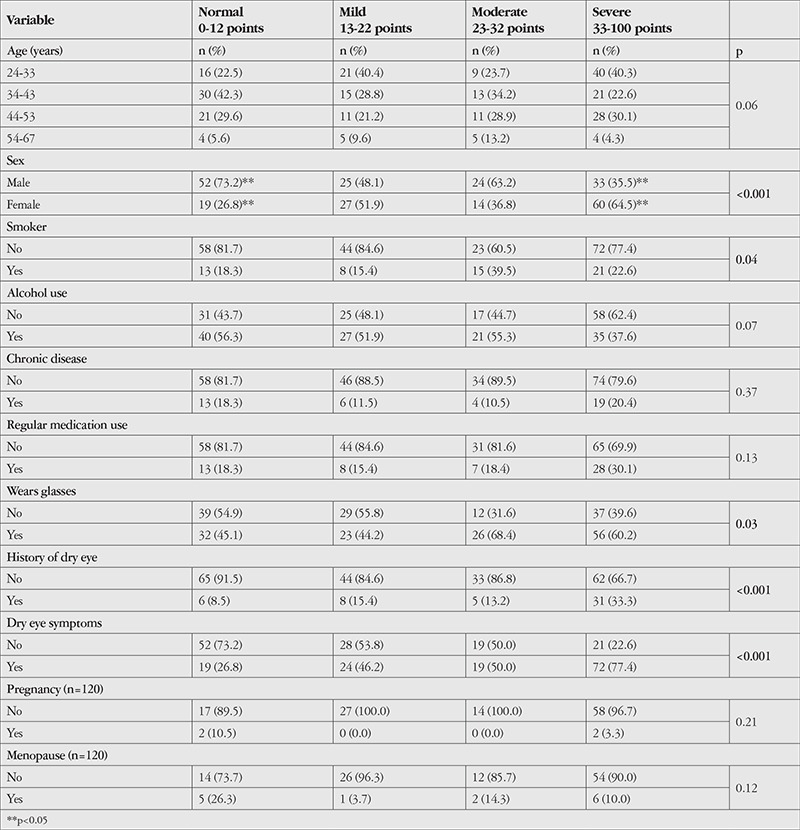
Distribution of demographic characteristics according to Ocular Surface Disease Index score categories
